# The Significance of Density Measurement and the Modified Bhalla and Reiff Scores in Predicting Exacerbations and Hospital Admissions in Cystic Fibrosis Patients

**DOI:** 10.3390/medicina61050808

**Published:** 2025-04-26

**Authors:** Oğuz Karcıoğlu, Selin Ardalı Düzgün

**Affiliations:** 1 Department of Chest Diseases, School of Medicine, Hacettepe University, Ankara 06100, Turkey; 2 Department of Radiology, School of Medicine, Hacettepe University, Ankara 06100, Turkey; selinardali@gmail.com

**Keywords:** bronchiectasis, computed tomography, exacerbation, pneumonia, score

## Abstract

*Background and Objective*: This study’s objective was to determine the impact of the percentage of lung tissue within the normal density range (PLND) on exacerbations and hospitalizations compared with the modified Bhalla and Reiff scores. We also investigated the effects of these measures on pulmonary function tests (PFTs). *Materials and Methods*: This retrospective analysis involved adult cystic fibrosis (CF) patients who had thoracic computed tomography (CT) while in a stable clinical condition. A dedicated radiologist analyzed CT images and conducted modified Bhalla, Reiff, and PLND assessments. We analyzed the exacerbations and hospitalizations in the year after the CT scan. We also examined PFTs at the time of the CT scan and one year later. *Results*: This study’s population consisted of 63 subjects (33 men), with a median age of 23.2 years. The median modified Bhalla score was 9.0 (IQR: 7.0–12.0), the median Reiff score was 11.0 (IQR: 8.0–15.0), and the median PLND was 79.4% (IQR: 74.5–82.0). The Bhalla score had the strongest relationship with both the number of exacerbations (*p* < 0.001, r: −0.559) and hospitalizations the following year (*p* < 0.001, r: −0.636), followed by the PLND score and the Reiff score. Youden’s index shows that the optimum cut-off values for hospitalization at ≤2 and >2 are 6.5 for the modified Bhalla score, 13.5 for the Reiff score, and 76.5% for the PLND. *Conclusions*: The measurement of PLND may serve as a predictor for exacerbation and hospitalization rates, aligning with the modified Bhalla and Reiff scores, and shows potential for application in follow-up assessments.

## 1. Introduction

Cystic fibrosis (CF), a multisystemic disorder, is caused by the presence of an improperly or non-functioning chloride channel, namely the cystic fibrosis transmembrane conductance regulator protein (CFTR), which is normally essential for the proper functioning of several organ systems. Although the autopsy findings of children who died of malnutrition led to the initial misname of “cystic fibrosis of the pancreas”, later research revealed that the lungs were the primary affected site and that lung involvement was the cause of death in most cases [1,2].

The retention of hyperviscous secretions across the airways triggers a persistent inflammatory response, either with or without bacterial colonization, resulting in structural damage in both the airways and lung parenchyma [3]. Pulmonary exacerbations (PExs), defined simply as an acute deterioration of the clinical condition, are often induced by certain microorganisms and result in some structural damage to the lungs or worsen the existing damage [4]. The vicious cycle of more damage causing more PExs and more PExs causing more damage also leads to a steady decline in pulmonary functions. Furthermore, non-infectious complications, such as pneumothorax and allergic bronchopulmonary aspergillosis (ABPA) may also contribute to the structural damage [5]. This extensive damage causes difficulties in evaluating radiological examinations, particularly X-rays.

Thorax computed tomography (CT) provides detailed information about the extent of the damage. It is possible to examine the presence of bronchiectasis, nodules, mucous plugging, and peribronchial thickening, as well as dilatation of bronchial arteries and an increase in pulmonary artery diameter indicative of pulmonary hypertension [6,7]. No specific recommendations exist regarding the role or frequency of serial CT examinations, but clinicians may utilize CT imaging to monitor disease or when X-rays fail to clarify the patient’s clinical presentation [8]. Various scoring systems have been developed to standardize lung damage, including Bhalla, BRICS, Reiff, bronchiectasis severity index (BSI), and Cystic fibrosis computerized tomography score (CF-CT) [9,10,11]. Although these scoring systems are used to determine the severity and extent of lung damage, the research on their effectiveness in predicting exacerbation frequency and changes in pulmonary function is limited.

Density measurement relies on the X-ray attenuation characteristics of tissue and is commonly used to ascertain the composition of pleural fluids [12,13,14]. It enables the assessment of the severity or extent of the lung abnormalities with decreased attenuation, such as emphysema or cystic changes, or increased attenuation, such as pulmonary fibrosis [15]. Moreover, it is possible to determine the percentage of lung tissue within the normal density range (PLND), despite its infrequent application and neglect.

Previously established scores are usually based on measuring the severity of bronchiectasis in CF patients. However, it is well known that CF lungs are not solely affected by bronchiectasis but also atelectasis, air trapping, cysts, fibrosis, etc. From this viewpoint, understanding the extent of normal lung tissue may be more significant than quantifying the extent of bronchiectasis in cystic fibrosis patients. The primary objective of this study was to investigate the impact of PLND and two commonly used scores, the modified Bhalla and the Reiff, on the exacerbation and hospitalization rates in adult patients with CF (pwCF). The secondary objective was to investigate the relationship between these scores and lung functions, as well as the impact of alterations in pulmonary functions.

## 2. Materials and Methods

### 2.1. Study Design

We performed a retrospective study to investigate the relationship between the severity of lung damage—determined by measuring the density and calculating the modified Bhalla and Reiff scores—and the exacerbation rates and pulmonary functions of adult pwCF. The study group consisted of patients aged 18 years and older who received follow-up care in the CF unit of Hacettepe University Adult Hospital. We included the patients who underwent thorax CT scans between July 2018 and July 2023, and had at least a follow-up period of one year. Therefore, the follow-up period ended in July 2024. We aimed to examine the CTs obtained during the stable period. Therefore, we excluded the thorax CT scans that were either acquired for a PEx of CF or obtained within one month before or after a PEx. If a patient underwent more than one CT scan during the study period, we preferred to record the first one to eliminate the risk of the subsequent CT being dependent on the previous CT.

**Figure 1:**
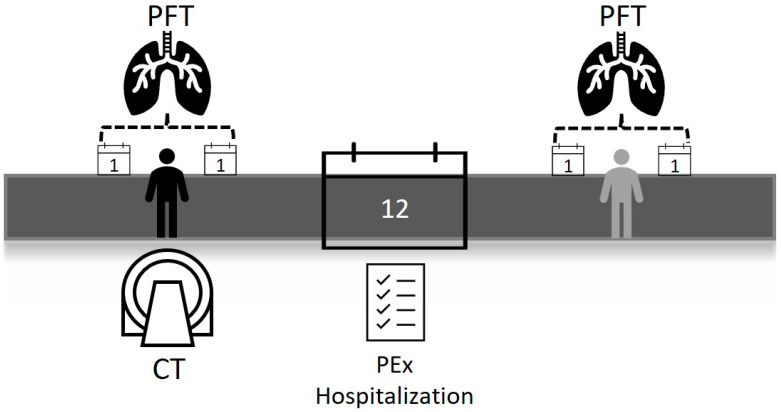
Timeline of pulmonary functions tests.

The initial pulmonary function test (PFT) was defined as the PFT results obtained either one month prior to or one month following the date of the CT scan. Pulmonary function tests performed one year after the date of the CT, again within a 2-month time frame, were also documented as the last tests (Figure 1). We separately recorded the total PExs and hospitalizations over a one-year period starting from the CT date. Demographic and other clinical data were obtained from the hospital registration system.

Informed consent was not required, as this was retrospective research utilizing the patients’ prior data from the hospital database.

### 2.2. CT Acquisition

CT acquisitions were performed using 16 (Sensation 16, Siemens Healthineers, Erlangen, Germany or Optima CT540, GE Healthcare, Milwaukee, WI, USA)- or 64-row (Somatom Perspective 64, Siemens Healthineers, Erlangen, Germany) multidetector CTs. Scans were performed during breath-hold, without intravenous contrast material administration using a tube voltage in the range of 100–120 kV, effective mAs in the range of 80–120, and a slice thickness in the range of of 3–5 mm. Images were reconstructed with a slice thickness between 1 and 1.5 mm.

### 2.3. CT Evaluation

CT scans were assessed using the modified Bhalla and Reiff scoring systems. In the modified Bhalla system, each parameter was assigned a score ranging from 0 to 3 for bronchiectasis severity, peribronchial thickening, bronchiectasis extent, mucous plugging extent, sacculation/abscesses, involved bronchial generation, bullae, and score ranging from 0 to 2 for emphysema, collapse/consolidation, and mosaic perfusion. The total score was subtracted from 25 to calculate the final Bhalla score [9].

Bronchiectasis severity was also evaluated using the Reiff score, which was calculated as the sum of scores ranging from 0 to 3 for each of the six lobes, with the lingula considered a separate lobe [11]. Also, a quantitative lung analysis was performed using CT Pulmo 3D (syngo.via version VB60_AHF07, Siemens Healthineers, Erlangen, Germany). After the automatic lung segmentation, parameters such as total lung volume, mean lung density, and PLND were calculated. Normal density was defined using attenuation values between −700 HU and −950 HU (Hounsfield Unit: HU) [16,17]. All measurements and semiquantitative CT scores were conducted by a thoracic radiologist with 9 years of experience in thoracic radiology who was blinded to the clinical information of the subjects.

### 2.4. Statistical Analysis

We conducted a univariate analysis for descriptives and presented the continuous variables either as mean (standard deviation) or median (IQR) regardless of the normality assumption. For categorical variables, the data were reported as frequency (%). We assessed the normality assumption utilizing the Shapiro–Wilk test, histograms, and boxplots. Spearman’s Rho was utilized for the correlation of non-parametric variables. Optimum cut-off points of the measurements were determined by considering the maximum value of Youden’s index, and 0.8 ≥ was accepted as excellent, and 0.7–0.8 as acceptable for discrimination according to the Hosmer–Lemeshow test. We performed ROC analyses of the modified Bhalla score, the Reiff score, and the PLND in relation to the number of hospitalizations. Statistical analysis was carried out using IBM SPSS version 23 (IBM SPSS Statistics for Windows, Version 23.0. Armonk, NY, USA: IBM Corp., released 2015). IBM SPSS Statistics for Windows, Version 23.0. Armonk, NY, USA: IBM Corp., were used. Statistical significance was determined at *p* < 0.05.

### 2.5. Ethics Statement

This study’s protocol adhered to the principles outlined in the Declaration of Helsinki. The Institutional Ethical Board approved this study’s protocol (SBA 24/890, 2024/14-27).

## 3. Results

This study’s population involved 63 individuals, with a median age of 23.2 years (IQR: 19.9–27.0). Of the patients, 33 (52.4%) were male. Three (4.7%) patients were on CFTR modulators at the time of the CT scan, and only one patient administered the drugs uninterruptedly. Four individuals began to use modulators within the year following the CT scan; however, none were able to use them continuously. The mean duration of drug use was 4.6 (±1.47) months among 6 patients who were able to use intermittent medication. Twenty-one (33.3%) subjects were on long-term oxygen therapy (LTOT). The majority suffered from exocrine pancreas insufficiency (55, 87.3%), followed by chronic liver disease (28, 44.4%) and CF-related diabetes mellitus (CFRD) (6, 9.5%). The median exacerbation and hospitalization numbers were 2 (IQR: 0–3) and 1 (IQR: 0–2), respectively. The median modified Bhalla score was 9.0 (IQR: 7.0–12.0) and the median Reiff score was 11.0 (IQR: 8.0–15.0). The mean total lung volume was 4974.5 ± 1116.9 milliliters, and the median lung density was −803 (IQR: −833–−771) HUs. The percentage and the volume of the lung parenchyma within the normal density range were 79.4% (IQR: 74.5–82.0) and 3859.2 ± 1072.4, respectively (Table 1).

Among the components of the Bhalla score, the highest points were obtained from the involvement of bronchial generations, the extension of bronchiectasis, and the severity of bronchiectasis. On the other hand, the least observed components were bullae and sacculations (Table 2).

The Bhalla score was the most significantly correlated variable with the number of exacerbations in the following year (*p* < 0.001, r: −0.559), followed by the Reiff score and the PLND (*p* < 0.001, r: 0.427; *p* < 0.002, r: −0.378, respectively). The Bhalla score strongly correlated with the number of hospitalizations in the subsequent year (*p* < 0.001, r: −0.636). The correlation between the number of hospitalizations and the PLND was slightly higher than the correlation with the Reiff score (*p* < 0.001, r = −0.567; *p* < 0.001, r = 0.512, respectively). The Bhalla score showed a strong correlation with first ppFEV1, and first ppFVC (*p* < 0.001, r = 0.736; *p* < 0.001, r = 0.732) followed by the PLND (*p* < 0.001, r = 0.665; *p* < 0.001, r = 0.706), and Reiff score (*p* < 0.001, r = −0.701; *p* < 0.001, r = −0.675) (Table 3). No statistically significant change was seen between the first and final PFT measurements; hence, a correlation analysis was not conducted.

**Figure 2:**
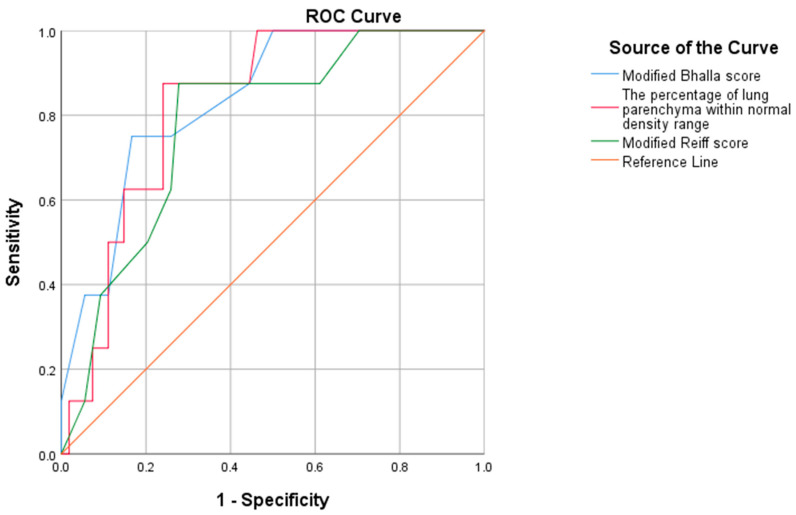
ROC analysis shows the AUC scores for hospitalization comparatively.

ROC analysis revealed no significant relationship between the number of exacerbations and the modified Bhalla score, the Reiff score, and the PLND. Youden’s index indicated that the optimal cut-off points for hospitalization at ≤2 and >2 were 6.5 for the modified Bhalla score, 13.5 for the Reiff score, and 76.5% for the PLND. ROC analysis showed that the AUC was 0.841 (0.710–0.966) for the modified Bhalla score, 0.785 (0.636–0.934) for the Reiff score, and 0.828 (0.713–0.944) for the PLND (Figure 2 and Table 4).

## 4. Discussion

The present study demonstrated correlations between the modified Bhalla score, the Reiff score, and the PLND with the number of exacerbations and hospitalizations in the year following the CT scan date. Specific scores from the modified Bhalla and Reiff scores, along with a threshold of the PLND, can predict whether patients will experience more than two hospitalizations. All three metrics showed a good correlation with the PFT results.

Although it is well known that CF causes structural damage to the lung parenchyma and airways, there is no consensus on the frequency of CT screening for both pediatric and adult pwCF due to radiation-related risk concerns [8]. To reduce the number of CT scans performed, many scoring systems have been proposed that predict subsequent exacerbations as well as PFT changes [18]. Bhalla et al. suggested scoring both the overall severity of structural damage and also individual pathologies, including the severity and extension of bronchiectasis, bronchial thickening, mucous plugging, and air trapping [19]. Later research confirmed that the modified Bhalla score may have a predictive value on quality of life, symptoms, and PFT [10,20]. We observed a strong correlation between the modified Bhalla score and ppFEV1 and ppFVC, as well as the number of hospitalizations, and a moderate correlation with the total number of exacerbations in our study. Low SFT measurements are known to be associated with both severe structural damage and increased risk of exacerbation. This study suggests that radiological examinations can be used to predict the risk of exacerbation, especially in periods when SFTs cannot be performed.

CF damages both the airways and lung parenchyma in affected individuals. Thus, it is not enough to merely summarize the damage by the existence of bronchiectasis. Determining the severity and the extent of bronchiectasis is also essential, as well as identifying pathologies that accompany or occur as a consequence of it, such as mucous plugging, air trapping, atelectasis, etc. Diab-Caceres et al. reported that, among the components of the modified Bhalla score, higher scores were obtained for the extension and severity of bronchiectasis and the generations of bronchial divisions, indicating the complete involvement of the bronchial tree [9]. Corroborating these findings, our CT examinations showed the same components to be predominant and severe, with bullae and sacculations being the least common. Our study utilized the CT scans of patients during a stable period, enabling us to exclude the findings associated with the acute exacerbation phase. This approach may elucidate the similarity of our results to those of the prospective study. Both investigations revealed that certain persistent pathologies were common in adult CF CTs, affecting the lungs entirely rather than the upper lobes, as previously believed [21].

One of the main determinants of the prognosis of CF is the frequency of exacerbations, with or without hospitalization [22,23]. More exacerbations result in a more significant reduction in pulmonary functions, leading to more symptoms [24]. It was shown that obtaining high severity scores from established tools examining severity using CT images means higher risks for both pwCF and patients with non-CF bronchiectasis [9,18,25,26]. In addition to assessing the presence and severity of pathologies, we believe it is crucial to determine the PLND. Therefore, we compared the impact of the modified Bhalla score established for CF, the Reiff score used for non-CF bronchiectasis, and the PLND on exacerbations and hospitalizations of patients with CF. The results reveal that, consistent with prior research, the modified Bhalla score demonstrates a good correlation with the number of hospitalizations and exacerbations. The Reiff score and the PLND exhibit lower correlations than the modified Bhalla score, yet are comparable to each other. Our results also show that all three tools have strong correlations with PFT measurements. In other words, it is as essential to know how healthy the lungs are as how damaged they are.

Semiquantitative scoring systems are vulnerable to subjective interpretation and may have limited sensitivity for identifying minor changes. Instead, quantitative CT analysis can provide a more accurate, objective, detailed, and time-saving assessment of lung structure with lower intra- and interobserver variability. On the other hand, it requires dedicated software, which may have a considerable cost. Since adult CF is a disease monitored in specific medical centers rather than universally, obtaining such software could allow for comparative use alongside semiquantitative scoring by radiologists.

Anticipating future exacerbations is essential for directing the care of cystic fibrosis. Although we already know that severe lung damage and low PFT measures are risk factors for later exacerbations, there is little knowledge regarding the extent of damage on CT that reliably predicts potential exacerbations [18,27,28]. It was argued that a modified Bhalla score of less than 15 points can predict the means of ≤2 PEx in the following year [9]. We could determine certain cut-off points in all three tools for the risk of subsequent hospitalizations, but not for PExs. This may be due to the fact that outpatient exacerbations (some of which are not real exacerbations) are determined by direct patient self-report and therefore may not contain the full true information.

Our study had some limitations. First of all, this was a retrospectively designed study with a limited number of individuals. Because the study encompassed the COVID-19 period, the majority of cases did not undergo follow-up PFTs; therefore, the impact of CT scores on changes in PFT measures could not be analyzed. Similarly, patients may have used oral antibiotics in cases of clinical deterioration without visiting their doctors due to COVID-19 concerns. It can be a handicap that the images were not evaluated by more than one radiologist. Bhalla also has an intrinsic limitation in detecting early changes. Finally, despite providing information on the use of modulators that influence exacerbation and hospitalization rates, we were unable to conduct an analysis on these drugs due to ongoing reimbursement challenges in our country. Even if our study population included a very limited number of individuals using modulators, it might be expected that these medications would have an effect on the number of exacerbations. Prospectively designed studies that categorize patients according to modulator use may contribute greatly to a more accurate use of CT assessment.

Including the real-life results of the adult pwCF is one of the strengths of this research. The inclusion of an examination of three different tools by an experienced radiologist blinded to the clinical data and PFT results adds strength to this study.

## 5. Conclusions

In conclusion, the concordance of the CT scoring tools and the density measures with exacerbation and hospitalization rates may allow us to follow-up patients through these tools, potentially decreasing the frequency of CT scans and associated radiation exposure. Additionally, they can provide insights into the pulmonary functions of individuals who are either unable or unwilling to perform pulmonary function tests (PFTs). Further prospectively designed research, including patients with and without modulators, conducted by more than one radiologist may provide information on their relationship with changes in PFT over time.

## Figures and Tables

**Table 1:** Demographics and measurements.

Variables, n = 63 Patients	
Age, median, median, (IQR)	23.2 (19.9–27.0)
Gender (male), n (%)	33 (52.4)
Mutations, n (%)	Homozygot F508del: 11 (17.5) Heterozygot F508del: 11 (17.5) Non-F508del: 31 (49.2) Unknown: 10 (15.9)
Modulator drug status (on), n, %	3 (4.7) at the time of CT scan 7 (11.1) any time during follow-up ***
Long-term oxygen therapy (on),	21 (33.3)
CFRD	6 (9.5)
Chronic liver disease	28 (44.4)
Chronic renal disease	6 (9.5)
Exocrin pancreas insufficiency	55 (87.3)
Initial FEV1, lt, median, (IQR) *	2.13 (1.14–2.99)
Initial ppFEV1, %, median, (IQR) *	55.5 (34.5–86.0)
Initial FVC, lt, median, (IQR) *	2.98 (1.84–4.02)
Initial ppFVC, %, median, (IQR) *	75.5 (49.5–94.5)
Initial FEV1/FVC, median, (IQR) *	69.1 (60.4–77.7)
Exacerbations in one year period, n, median, (IQR)	2 (0–3)
Hospitalization in one year period, n, median, (IQR)	1 (0–2)
Last FEV1, lt, median, (IQR) **	2.41 (1.50–3.24)
Last ppFEV1,%, median, (IQR) **	60.5 (43.0–87.0)
Last FVC, lt, median, (IQR) **	3.33 (2.15–4.61)
Last ppFVC, %, median, (IQR) **	88.5 (55.0–99.5)
Last FEV1/FVC, median, (IQR) **	70.1 (60.1–80.2)
Modified Bhalla score, median, IQR	9.0 (7.0–12.0)
Modified Reiff score, median, IQR	11.0 (8.0–15.0)
Total volume, mililiters, mean ± SD	4974.5 ± 1116.9
Density, HU, median, (IQR)	−803 (−830–−776)
Volume of lung parenchyma within normal density, mililiters, mean ± SD	3859.2 ± 1072.4
Percentage of lung parenchyma within normal density, %, median, (IQR)	79.4 (74.5–82.0)

n: number, IQR: interquartile range, CFRD: cystic fibrosis-related diabetes mellitus, FEV1: forced expiratory volume in second 1, ppFEV1: percent-predicted forced expiratory volume in second 1, FVC: forced vital capacity, ppFVC: percent-predicted forced vital capacity, SD: standard deviation. * First PFT measurements were performed on 52 patients. ** Last PFT measurements were performed on 36 patients. *** Only one patient was able to use the drugs continuously. Six of them had F508del mutations and the other patient had R1070Q.

**Table 2:** Individual components of the modified Bhalla score.

	0	1	2	3
Severity of bronchiectasis	1 (1.6%)	5 (7.9%)	13 (20.6%)	44 (69.8%)
Thickening	2 (3.2%)	25 (39.7%)	29 (46.0%)	7 (11.1%)
Extension of bronchiectasis	1 (1.6%)	3 (4.8%)	9 (14.3%)	50 (79.4%)
Mucous plugging	3 (4.8%)	4 (6.3%)	18 (28.6%)	38 (60.3%)
Sacculations	31 (49.2%)	22 (34.9%)	8 (12.7%)	2 (3.2%)
Involvement of bronchial generations	1 (1.6%)	0	1 (1.6%)	61 (96.8%)
Bullae	41 (65.1%)	8 (12.7%)	12 (19.0%)	2 (3.2%)
Air trapping	20 (31.7%)	23 (36.5%)	20 (31.7%)	
Atelectasis	16 (25.4%)	25 (39.7%)	22 (34.9%)	

**Table 3:** Correlation between modified Bhalla and Reiff scores, density measurements and exacerbation numbers, and hospitalization numbers and PFT.

	Exacerbations	Hospitalizations	First FEV1	First ppFEV1	First FVC	First ppFVC	First FEV1/FVC
Variables							
Modified Bhalla score	−0.559 **	−0.636 **	0.685 **	0.736 **	0.669 **	0.732 **	0.396 **
Severity of bronchiectasis	0.394 **	0.333 **	−0.457 **	−0.406 **	−0.438 **	−0.361 **	−0.278 *
Thickening	0.542 **	0.492 **	−0.479 **	−0.504 **	−0.497 **	−0.534 **	−0.150
Extension of bronchiectasis	0.466 **	0.360 **	−0.568 **	−0.639 **	−0.493 **	−0.604 **	−0.504 **
Mucous plugging	0.520 **	0.456 **	−0.566 **	−0.593 **	−0.532 **	−0.584 **	−0.404 **
Sacculations	0.430 **	0.541 **	−0.599 **	−0.563 **	−0.610 **	−0.558 **	−0.276 *
Involvement of bronchial generations	0.239	0.171	−0.326 *	−0.289 *	−0.326 *	−0.299 *	−0.062
Bullae	0.095	0.383 **	−0.546 **	−0.603 **	−0.571 **	−0.640 **	−0.256
Air trapping	0.328 **	0.386 **	−0.625 **	−0.686 **	−0.560 **	−0.636 **	−0.532 **
Atelectasis	0.162	0.265 *	−0.274 *	−0.227	−0.328 *	−0.241	0.019
Reiff score	0.427 **	0.512 **	−0.728 **	−0.701 **	−0.705 **	−0.675 **	−0.486 **
Total LV	0.014	−0.134	0.351 *	0.173	0.453 **	0.192	−0.239
Mean density	0.231	0.405 **	−0.404 **	−0.419 **	−0.455 **	−0.452 **	0.039
Percentage within normal density range(%)	−0.378 **	−0.567 **	0.661 **	0.665 **	0.696 **	0.706 **	0.194
Total volume within normal density range	−0.092	−0.280 *	0.476 **	0.328 *	0.573 **	0.356 **	−0.148

* Correlation is significant at the 0.01 level (2-tailed). ** Correlation is significant at the 0.05 level (2-tailed).

**Table 4:** Cut-off points for predicting number of hospitalizations.

Variables	AUC (95% CI)	*p*-Value	Optimal Cut-Off Point *	Sensitivity	Specificity
Modified Bhalla score	0.841 (0.710–0.966)	0.002	6.5	0.75	0.836
Modified Reiff score	0.785 (0.636–0.934)	0.01	13.5	0.875	0.727
The percantage of normal parenchyma within normal lung density range	0.828 (0.713–0.944)	0.003	76.5	0.875	0.764

* Optimum cut-off point is specified by considering the maximum value of Youden’s index.

## Data Availability

The data presented in this study are available in case of a reasonable request from the corresponding authors.

## Abbreviations

The following abbreviations are used in this manuscript:

CF	Cystic fibrosis
pwCF	Patient with cystic fibrosis
CFTR	Cystic fibrosis transmembrane conductance regulator protein
CFRD	Cystic fibrosis-related diabetes
CT	Computed tomography
PFTs	Pulmonary function tests
PLND	Percentage of lung tissue within the normal density range
PEx	Pulmonary exacerbation
ABPA	Allergic bronchopulmonary aspergillosis
BSI	Bronchiectasis severity index
CF-CT	Cystic fibrosis computerized tomography score
